# Effect of plant root symbionts on performance of native woody species in competition with an invasive grass in multispecies microcosms

**DOI:** 10.1002/ece3.4397

**Published:** 2018-08-02

**Authors:** Christina Birnbaum, Tim K. Morald, Mark Tibbett, Richard G. Bennett, Rachel J. Standish

**Affiliations:** ^1^ Environmental and Conservation Sciences School of Veterinary and Life Sciences Murdoch University Murdoch WA Australia; ^2^ School of Biological Sciences The University of Western Australia Crawley WA Australia; ^3^ Centre for Agri‐Environmental Research & Soil Research Centre School of Agriculture, Policy and Development University of Reading Reading UK; ^4^ Centre for Plant Genetics and Breeding The University of Western Australia Crawley WA Australia

**Keywords:** invasion, legumes, old‐field restoration, plant–soil interactions, symbiosis

## Abstract

The majority of terrestrial plants form mutualistic associations with arbuscular mycorrhizal fungi (AMF) and rhizobia (i.e., nitrogen‐fixing bacteria). Understanding these associations has important implications for ecological theory and for restoration practice. Here, we tested whether the presence of AMF and rhizobia influences the performance of native woody plants invaded by a non‐native grass in experimental microcosms. We planted eight plant species (i.e., *Acacia acuminata*,* A. microbotrya*,* Eucalyptus loxophleba subsp. loxophleba, E. astringens, Calothamnus quadrifidus*,* Callistemon phoeniceus*,* Hakea lissocarpha* and *H. prostrata*) in microcosms of field‐conditioned soil with and without addition of AMF and rhizobia in a fully factorial experimental design. After seedling establishment, we seeded half the microcosms with an invasive grass *Bromus diandrus*. We measured shoot and root biomass of native plants and *Bromus*, and on roots, the percentage colonization by AMF, number of rhizobia‐forming nodules and number of proteaceous root clusters. We found no effect of plant root symbionts or *Bromus* addition on performance of myrtaceous, and as predicted, proteaceous species as they rely little or not at all on AMF and rhizobia. Soil treatments with AMF and rhizobia had a strong positive effect (i.e., larger biomass) on native legumes (*A*. *microbotrya* and *A. acuminata*). However, the beneficial effect of root symbionts on legumes became negative (i.e., lower biomass and less nodules) if *Bromus* was present, especially for one legume, i.e., *A. acuminata*, suggesting a disruptive effect of the invader on the mutualism. We also found a stimulating effect of *Bromus* on root nodule production in *A*. *microbotrya* and AMF colonization in *A. acuminata* which could be indicative of legumes’ increased resource acquisition requirement, i.e., for nitrogen and phosphorus, respectively, in response to the *Bromus* addition. We have demonstrated the importance of measuring belowground effects because the aboveground effects gave limited indication of the effects occurring belowground.

## 1 INTRODUCTION

Mutualistic associations between soil organisms and plants are common in nature, particularly those involving mycorrhizal fungi and rhizobia (Schupp, Jordano, & Gómez, 2017). These plant symbionts can strongly influence dynamics of plant communities. For example, rhizobia have been reported to contribute to aboveground plant productivity and plant community evenness (Barrett, Bever, Bissett, & Thrall, 2015; van der Heijden et al., 2006). Additionally, arbuscular mycorrhizal fungi (AMF) have been shown to determine plant species diversity (Hiiesalu et al., 2014; Teste et al., 2017) and affect interspecific competition (Fonseca, Dias, Carolino, França, & Cruz, 2017; Lin, McCormack, & Guo, 2015) and plant productivity (Bauer, Blumenthal, Miller, Ferguson, & Reynolds, 2017). Indeed, Klironomos et al. (2011) have suggested that mycorrhizal associations could be as important as herbivory or competition for structuring plant communities.

In recent years, soil microbial communities have widely been acknowledged to contribute to the success of invasive species (Callaway, Bedmar, Reinhart, Silvan, & Klironomos, 2011; Callaway, Thelen, Rodriguez, & Holben, 2004; Inderjit & van der Putten, 2010; Reinhart & Callaway, 2006; van der Putten et al., 2009) and there is some evidence for soil organisms being important for resistance to invasion. For example, soil organisms provided biotic resistance to native plants against invasive *Potentilla* (Callaway, Montesinos, Williams, & Maron, 2013). Additionally, biotic resistance conferred by soil pathogens was reported by Knevel, Lans, Menting, Hertling, and van der Putten (2004) for invasive dune grass *Ammophila arenaria* in South Africa. Thus, soil microbes may enhance biotic resistance of plant communities to weed invasion and in turn affect community structure.

The role of plant root symbionts in invasion success has received increasing attention (Birnbaum, Bissett, Thrall, & Leishman, 2016; Klock, Barrett, Thrall, & Harms, 2015; Shelby et al., 2016; Stampe & Daehler, 2003; Wandrag, Sheppard, Duncan, & Hulme, 2013). An absence of ectomycorrhizal fungi has been reported to hinder the invasion success of exotic pines (Hayward, Horton, Pauchard, & Nuñez, 2015; Nuñez, Horton, & Simberloff, 2009). Other authors have suggested that invasive species have higher AMF colonization rates which may contribute to their higher total biomass compared with native species, and subsequently AMF may contribute to their invasion success (Paudel, Baer, & Battaglia, 2014). Rhizobia have also been suggested to facilitate legume establishment success in the introduced (invasive) ranges (Rodríguez‐Echeverria, 2010). Overall, these and other studies show that plant root symbionts play important roles as gate‐keepers to plant community membership.

Understanding the contribution of plant root symbionts, their interactions, and their linkages to plants as determinants of plant community structure has important implications for ecological theory (Lambers et al., 2017). Beyond these theoretical implications, there are important practical outcomes too, i.e., this knowledge could help to refine frameworks for ecological restoration and could inform management practises more generally (Birnbaum, Bradshaw, Ruthrof, & Fontaine, 2017; Kardol & Wardle, 2010). For example, in old‐field restoration, often the aim is to overcome the resistance of the resident weedy community in order to establish a target community that is, in turn, resistant to reinvasion by the weedy species. Overcoming the resistance of the resident weedy community might be challenging if it is coupled with land‐use legacies in soil (Kulmatiski, Beard, Stevens, & Cobbold, 2008) or soil conditioning by invasive species (Hawkes, Wren, Herman, & Firestone, 2005; Vink et al., 2017). Emerging evidence suggests that better understanding of land‐use legacies on plants and their associated soil microbial communities could inform old‐field restoration (e.g., Hannula et al., 2017; Strickland et al., 2017).

Here, the primary aim was to test the role of plant root symbionts in plant species coexistence and response to plant invasion using experimental microcosms. Our experimental design was informed by the Ridgefield Multiple Ecosystem Services Experiment (henceforth the Ridgefield Experiment) established on an old‐field in southwestern Australia (Perring et al., 2012). The Ridgefield Experiment was established to determine the relationship between the species diversity of woody plants and ecosystem functions in restoration, and additionally, the delivery of ecosystem services in the context of global change (e.g., N deposition, biological invasion; Perring et al., 2012). The microcosm experiment was designed to complement the questions being tested by the Ridgefield Experiment and uses soils, native plants from three different families (i.e., Fabaceae, Myrtaceae, and Proteacea), fungi and rhizobia from the vicinity of this field experiment. Specifically, we hypothesized that the presence of AMF and rhizobia would: a) positively influence the competitive outcomes among native woody plant species from Fabaceae that form associations with both symbionts and Myrtaceae that form association with AMF over Proteaceae that do not form associations with these symbionts and b) be beneficial to Fabaceae and Myrtaceae in resisting the *Bromus diandrus* invasion, whereas not affect response of Proteaceae to invasion in our experimental microcosms.

## 2 MATERIALS AND METHODS

### 2.1 Study system

The Ridgefield Experiment (32°29′S, 116°58′E) includes native plant species *Acacia acuminata* Benth. and *A. microbotrya* Benth. (Fabaceae); *Eucalyptus loxophleba* Benth. *subsp. loxophleba* (henceforth *E. loxophleba*), *E. astringens* (Maiden) Maiden, *Calothamnus quadrifidus* R.Br., and *Callistemon phoeniceus* Lindl. (Myrtaceae); and *Hakea lissocarpha* R.Br. and *H. prostrata* R.Br. (Proteaceae). We used all eight species in our experiment. The *Acacia* species associate with both AMF and rhizobia. The four myrtaceous species associate with AMF but not rhizobia, and the two *Hakea* (proteaceous) species associate with neither mutualist, but instead form cluster roots (Supporting Information Table S1). AMF associations are visible in stained roots under a microscope, roots colonized by rhizobia develop nodules visible with the naked eye, and cluster roots are bottlebrush‐like structures also visible with the naked eye. Cluster roots “mine” phosphorus fixed as insoluble inorganic phosphates (e.g., iron phosphate and rock phosphate) in phosphorus‐impoverished ancient landscapes (Lambers, Raven, Shaver, & Smith, 2008).

The invasive species *Bromus diandrus* Roth (Poaceae) occurs in and around the Ridgefield Experiment. It is a Mediterranean annual C3 grass introduced to Australia circa 1875 from the Mediterranean Basin as a contaminant of crop seeds or wool (Brown & Bettink, 2009). It is widespread in Australia, California (USA), Chile, and New Zealand (Kleemann & Gill, 2009; Parsons & Moldenke, 1975; Tozer, Marshall, Sedcole, & Edwards, 2007). In southern Australia, *Bromus diandrus* completes its full life cycle during the winter wet season and before the onset of the summer drought (Gill & Blacklow, 1985). At the Ridgefield Experiment, seed germination and seedling establishment occurs in the winter wet season (May–July), and flowering and seeding occurs in spring (Sept–Nov; R. J. Standish, pers. obs.). Seeds tend to germinate within 1 month of shedding from the parent plant (Harradine, 1986), and few seeds are stored in the soil (Standish, Stokes, Tibbett, & Hobbs, 2007). Seed germination is rapid (i.e., within 40 hours after seeds imbibe water; Gill & Blacklow, 1985). Growing season length varies between 92 and 107 days depending on wet season length (Gill & Blacklow, 1985). *Bromus diandrus* is known to associate with AMF including *Glomus tenue* (Greenall) I.R. Hall (Hilbig & Allen, 2015; Sigüenza, Corkidi, & Allen, 2006). *Glomus tenue* is present in a wide range of soils including agricultural soils of southwestern Australia (Abbott & Robson, 1977; Gucwa‐Przepióra, Blaszkowski, Kurtyka, Malkowski, & Malkowski, 2013; Orchard, Standish, Nicol, Gupta, & Ryan, 2016). Lastly, *Bromus diandrus* does not associate with rhizobia.

### 2.2 Experimental design

To test whether the performance of native plant species depended on the presence/absence of their plant root symbiont (i.e., AMF and rhizobia), we had four soil treatments (+AMF+Rhiz, +AMF−Rhiz, −AMF+Rhiz, and −AMF−Rhiz) with and without the invasive grass *B. diandrus*, each replicated four times (*n *=* *4 × 2 × 4 = 32 microcosms). We predicted that the two *Acacia* species and four myrtaceous species would perform better with access to their plant root symbiont/s (i.e., in the +AMF+Rhiz and the +AMF−Rhiz soil treatments, respectively) and that the two proteaceous species would perform better in the −AMF−Rhiz soil treatment. The −AMF+Rhiz treatment was included to compare the performance of the *Acacia* species with access to one and both plant root symbionts. Microcosms were laid out in a completely randomized block design (block = replicate).

Soil for the experiment was collected from an area adjacent to the Ridgefield Experiment in April 2011 and steam pasteurized (i.e., 80–90°C twice, 24 hr apart) within 3 weeks of collection. The soil was dried in a clean soil‐drying room, then bagged, and stored for less than a month in a sterile potting room at the Plant Growth Facility, the University of Western Australia (UWA).

Seeds of native species were sourced from wild populations across the wheatbelt of southwestern Australia, one population per species. Seeds of the invasive grass *Bromus diandrus* were collected from Ridgefield in Spring 2010. Prior to germination, seeds were surface‐sterilized in 70% ethanol for 60 s and then in 4% NaHClO_4_ for 30 s and rinsed in sterile DI (deionized) water six times to avoid contaminating the experiment with other microbes. *Acacia* seeds were boiled for 30 s to break dormancy. All seeds were germinated on moist filter papers in sealed Petri dishes kept in the dark and in a constant temperature (15°C) room until cotyledon stage.

In June 2011, seedlings of uniform size were transplanted to tubs of 35 cm × 35 cm (henceforth microcosm) filled with 24 kg pasteurized soil from the Ridgefield Experiment. Sixteen native woody seedlings (two individuals per eight species) were randomly planted into the microcosms 7.5 cm apart and 5.5 cm from side of the tub in a 4 × 4 grid (Supporting Information Figure S1A). Alkathene polyethylene beads (Qenos Pty Ltd, Altona, Victoria, Australia) were added to the soil surface within each microcosm (250 ml per microcosm) to help prevent airborne spores of AMF and rhizobia contaminating microcosms without AMF and rhizobia. Dead seedlings were replaced with live seedlings in the first 12 weeks of the experiment. Eight seedlings (of 512 in total) died after 12 weeks and were not replaced; these were three *E. loxophleba*, three *C. quadrifidus* and two *C. phoeniceus* in seven different microcosms. At 12 weeks, when native seedlings were established, 256 seeds of *B. diandrus* (henceforth *Bromus*) were added to half the microcosms. The sowing distance between *Bromus* seeds was 2 cm, mimicking field densities (Supporting Information Figure S1B). *Bromus* seedlings started to appear 4 days after sowing. Seed germination and seedling establishment was high; on average (±1*SE*), 238 ± 7 *Bromus* seedlings were harvested from the microcosms.

Microcosms were watered to field capacity with boiled and cooled deionized water weekly and then biweekly as the seedlings became larger. We used a gantry crane to lift microcosms onto a balance for weighing and watering. Nutrients were not added to the experiment to avoid interference and confounding plant growth benefits of AMF and rhizobia. The soil used in the experiment here was collected from a plot in the Ridgefield Experiment with these soil chemical characteristics: mean total N (%) 0.165 (±0.005), total P (mg/kg) 253.6 (±9.77), available (Colwell) P (mg P/kg) 39.77 (±1.92), ammonium (mg/kg) 2.59 (±0.12), and organic C (%) 1.73 (±0.05) (Perring et al., 2012).

### 2.3 AMF and rhizobia inoculation

We predicted that AMF and rhizobia will be beneficial to Fabaceae and Myrtaceae in resisting the *Bromus* invasion and not affect Proteaceae response to invasion in our experimental microcosms. We had access to AMF and rhizobia from agricultural soils in Western Australia. The AMF was *Scutellospora calospora* (Nicolson & Gerdermann) Walker & Sanders (Gigasporaceae) spores originally sourced from P‐fertilized pasture in Badgingarra, Western Australia. *Scutellospora calospora* was subsequently maintained and proliferated via pot cultures (grown in pasteurized washed river sand with leeks (*Allium ampeloprasum* L.) as host plants in glasshouses at UWA. This AMF species has been found in old‐fields elsewhere including those at Cedar Creek USA (Johnson, Zak, Tilman, & Pfleger, 1991). *Scutellospora calospora* can form associations with jarrah (Eucalyptus marginata) seedlings (Kariman, Barker, Finnegan, & Tibbett, 2014), and we expected it would form associations with the four myrtaceous species and two *Acacia* species planted in our experiment. Rhizobia tend to be generalists in their associations with legumes (incl. *Acacia*) and are resident in most soils (Birnbaum, Bissett, Teste, & Laliberté, 2018; Birnbaum et al., 2016; Lafay & Burdon, 1998; Leary, Singleton, Scowcroft, & Borthakur, 2006; Thrall, Burdon, & Woods, 2000).

To prepare rhizobial inoculum, we inoculated each microcosm with a rhizobial suspension prepared from rhizobial strains known to associate with *Acacia* species in the field. This rhizobial inoculum was prepared by first isolating several different rhizobia from field collected nodules of *Acacia* species growing near Dwellingup, Western Australia. To select the most suitable strain among these rhizobia, the *Acacia* species used in this experiment were inoculated and, based on nodule counts, the strains forming the greatest number of nodules were selected to prepare individual rhizobial inoculums. One inoculum, that is, one strain of rhizobia, was used to inoculate the microcosms. Rhizobial inoculum was prepared by mixing ~700 ml yeast mannitol agar broth containing approximately 1 × 10^6^ live rhizobia cells with 850 ml of sterile water. For the treatment without rhizobia, ~700 ml of dead, i.e., autoclaved, rhizobial inoculum was mixed with ~800 ml of sterile water. In June 2011, 50 ml of live or dead rhizobial inoculum was syringed over the soil surface of microcosms.

For the AMF inoculum, pot‐cultured *S. calospora* inoculum (henceforth AMF inoculum) consisting of spores, hyphae, and colonized leek roots was mixed at a rate of 1:9 with soil (1 part inoculum: 9 parts pasteurized field soil) and then used in the AMF microcosms. To account for non‐AMF soil microorganisms likely to be present in the AMF inoculum (Nazeri, Lambers, Tibbett, & Ryan, 2013), an amount of inoculum equal to that used for the AMF treatment was vigorously mixed with DI water and then passed through a series of sterile autoclaved sieves from 2 mm to 8 μm, the finest mesh size preventing the smallest spores from entering the resultant filtrate. For the microcosms that did not receive the AMF inoculum (i.e., −AMF+Rhiz and −AMF−Rhiz), 125 ml of filtrate (~1/16 of what was produced) was evenly applied to each microcosm prior to planting seedlings. This filtrate contained the previously sieved, but autoclaved AMF inoculum (i.e., 1 part autoclaved inoculum: 9 parts pasteurized field soil). By autoclaving the inoculum, we prevented transferring AMF to non‐AMF treatments.

### 2.4 Plant growth and harvest

Microcosms were grown in the glasshouse from 24th June 2011. The mean glasshouse temperature during the experiment was 17.2°C (range 8.3–34.3°C), the relative humidity was 52.1%, and the glasshouse permitted ambient light. The growing conditions in the glasshouse were similar to those seedlings that would experience during establishment in the field (Perring et al., 2012). We invaded microcosms with *Bromus* on 4th to 6th October 2011. Microcosms were dense with plants by late October, and water‐use per microcosm increased to ~0.5 L per day with increasing air temperatures in the glasshouse. Concerned that we would not be able to disentangle roots of individual plants in the microcosms, we began harvesting microcosms, block by block, on 31st October and finished on 14th November 2011 (Supporting Information Figure S2A). The tradeoff with this decision was that *Bromus* seedlings completed only ~half of their life cycle and were shorter than the native seedlings at harvest (Supporting Information Figure S2B,C). Therefore, *Bromus* did not reduce light availability to native seedlings in the microcosms as it does in old‐fields elsewhere (e.g., California; Molinari & D'Antonio, 2014). However, our primary interest was belowground because competition between weeds and native woody seedlings is for water rather than light in our study system (Standish et al., 2007). The density of plants in the microcosms meant there was potential for belowground competition between young *Bromus* and the native seedlings (Supporting Information Figure S2B,C). Our measure of invasion resistance is relevant to native seedling establishment on weedy old‐fields where water is limiting (Cramer, Hobbs, & Standish, 2008).

At harvest, shoots and roots were separated and soil was washed from the roots. Root nodules and cluster roots were counted on each plant. A subsample of fine roots were cut and stored in 50% (v/v) ethanol pending assessment of AMF colonization. The root subsamples were cleared with 10% KOH for 1 week at room temperature, then rinsed with DI water, and acidified with 0.1 mol/L HCl. Thereafter, roots were stained in a 5% (v/v) blank ink vinegar solution (Vierheilig, Coughlan, Wyss, & Piché, 1998). Percentage AMF colonization of roots was estimated using the line intercept method (Giovannetti & Mosse, 1980). Shoots and remaining roots were dried for 48 hr at 60°C and weighed.

### 2.5 Data analysis

The response variables were above‐ and belowground biomass for all plant species; the number of root nodules on the two *Acacia* species; the percentage root length colonized by AMF for *Acacia* species, four myrtaceous species and *Bromus*; and the number of clusters for the two *Hakea* species (Proteaceae). Main and interactive effects of fixed factors and *Bromus* (two levels: present or absent) and soil treatment (four levels: +AMF+Rhiz, +AMF−Rhiz, −AMF+Rhiz and −AMF−Rhiz) were tested using linear mixed‐effects models with Type III SS and the lmer () function. Block was set as random factor in all models. Data and residuals were visually inspected for homogeneity and normality assumptions of linear models. Data were LN‐, log10‐, or square‐root‐transformed in cases where these assumptions were not met. Nodule data for *Acacia microbotrya* did not conform to normality with transformation and instead were analyzed using glmer () function with family = “poisson.” Tukey's post hoc tests were performed for pairwise analyses between the treatments. All data were analyzed and plotted in R programming language (version 3.4.3) (R Core Team, 2018) using the “lme4” (Bates, Mächler, Bolker, & Walker, 2014), “lmerTest” (Kuznetsova, Brockhoff, & Christensen, 2017), “Tidyverse” (H. Wickham, 2017), “ggplot2” (H. Wickham, 2009), “Rmisc” (Hope, 2013), and “multcomp” (Hothorn, Bretz, & Westfall, 2008) packages.

We derived mycorrhizal dependency values for the species with mycorrhizal associations. Dependency values were defined as the relationship between the dry mass of plants inoculated with mycorrhiza and the dry mass of uninoculated plants; a dependency value of >0 indicated that plants benefit from the association (after Gerdemann, 1975). The mycorrhizal dependency (MD) was calculated for each species using the formula MD (%) = (DW of mycorrhizal plant − DW of noninoculated plant)/DW of mycorrhizal plant × 100 (Kumar, Sharma, & Mishra, 2010), where DW is shoot dry weight. For each species, differences in DW when plants were grown with AMF and without AMF inoculation were analyzed using independent 2‐group *t*‐test.

### 2.6 Power analysis

We conducted post hoc power analyses of the linear mixed‐effects models using the “pwr” package in R (Champely, 2017). The recommended effect sizes for these analyses were: small (*f*_2_ = 0.02), medium (*f*_2_ = 0.15), and large (*f*_2_ **= 0.35) (Cohen 1977). The alpha level used for these analyses was *p *<* *0.05. The statistical power of our models was 0.08 to detect a small effect, 0.37 to detect a medium effect and 0.75 to detect a large effect. Thus, these power analyses suggested effect sizes needed to be large to be statistically significant in our models. Despite the power limitation, 13 of the 29 models we ran in total showed a statistically significant response to one or both treatments (Supporting Information Table S2). Due to a high number of models run, it is plausible that two models were significant by chance. Recall too that we anticipated no effect of AMF and rhizobia soil treatments on the biomass of Proteaceae (i.e., 4 of 29 models).

## 3 RESULTS

### 3.1 Plant biomass

**Figure 1:**
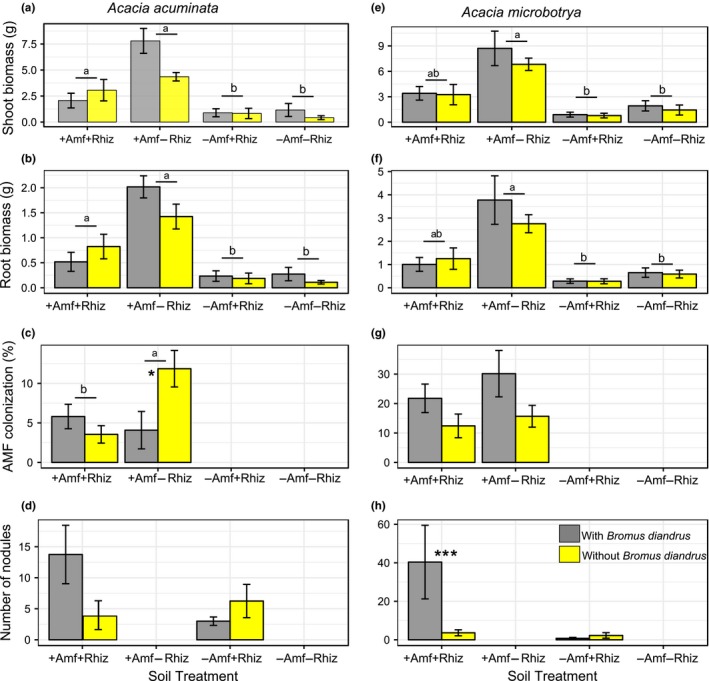
Plant performance data for *Acacia acuminata* (a‐d) and *A. microbotrya* (e‐h) (Fabaceae). Data are means ± *SE*. Nodules on roots indicate rhizobial associations. Different letters (underlined) above the bars indicate significant differences between treatment means at *p* < 0.05. Asterisks (***) indicate a statistically significant difference between *Bromus* treatments within soil treatments at *p *<* *0.001 [Colour figure can be viewed at http://wileyonlinelibrary.com]

**Figure 2:**
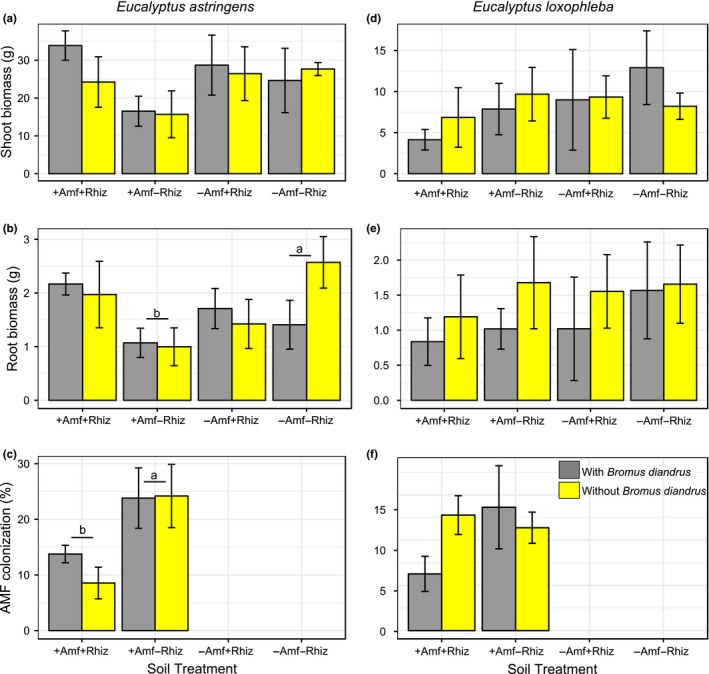
Plant performance data for *Eucalyptus astringens* (a‐c) and *E. loxophleba* (d‐f) (Myrtaceae). Data are means ± *SE*. Different letters (underlined) above the bars indicate significant differences between treatment means at *p *<* *0.05 [Colour figure can be viewed at http://wileyonlinelibrary.com]

**Figure 3:**
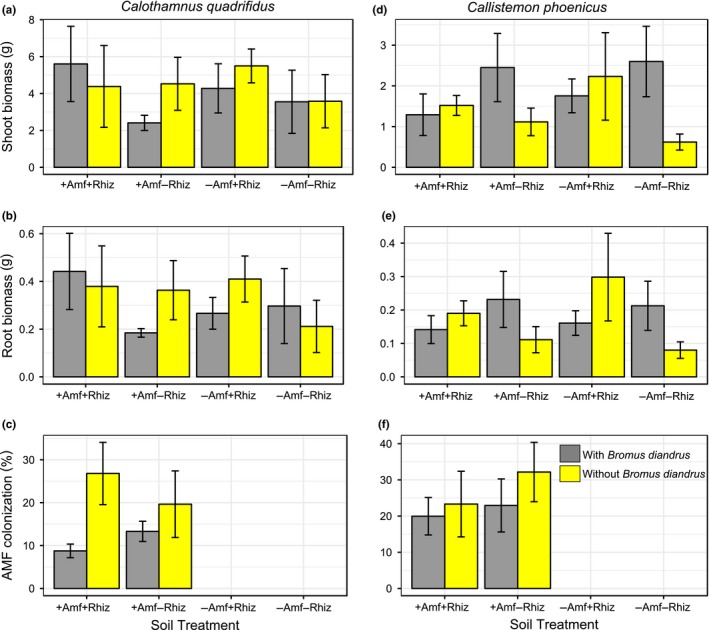
Plant performance data for *Calothamnus quadrifidus* (a‐c) and *Callistemon phoenicus* (d‐f) (Myrtaceae). Data are means ± *SE*. There were no significant differences between treatment means at *p *<* *0.05 [Colour figure can be viewed at http://wileyonlinelibrary.com]

**Figure 4:**
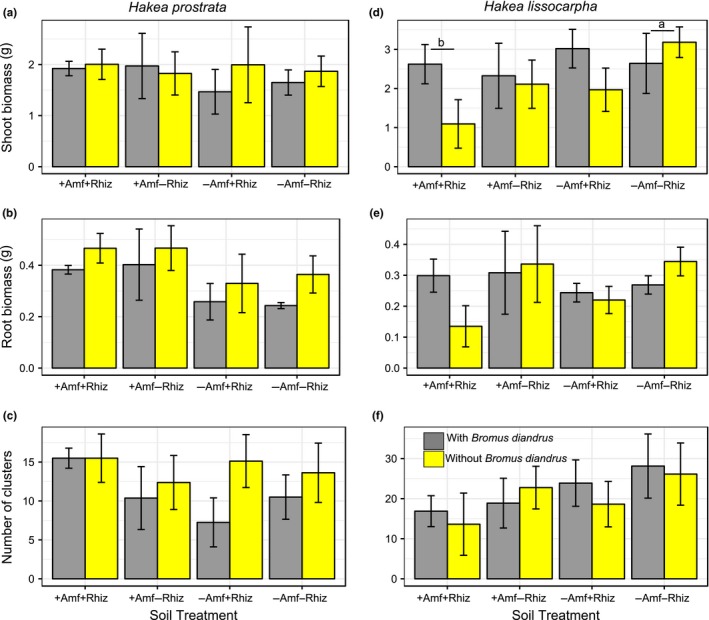
Plant performance data for *Hakea prostrata* (a‐c) and *H. lissocarpha* (d‐f) (Proteaceae). Data are means ± *SE*. Clusters are bottlebrush‐like structures on roots. Different letters (underlined) above the bars indicate significant differences between treatment means at *p *<* *0.05 [Colour figure can be viewed at http://wileyonlinelibrary.com]

**Figure 5:**
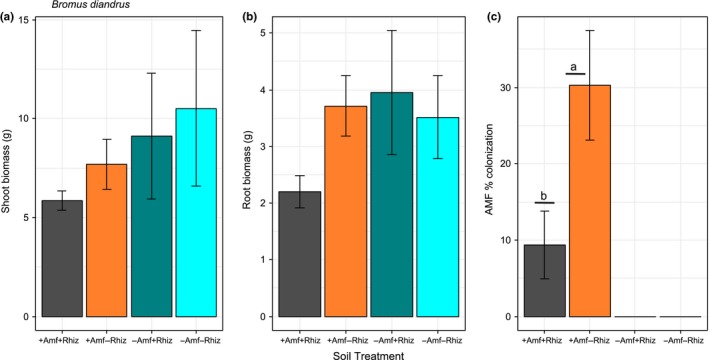
Plant performance data for *Bromus diandrus* (a‐c) (Poaceae). Data are means ± *SE*. Different letters indicate significant differences between treatment means at *p *<* *0.05 [Colour figure can be viewed at http://wileyonlinelibrary.com]

We hypothesized that the two *Acacia* species and four myrtaceous species would perform better with access to their plant root symbiont/s (i.e., in the +AMF+Rhiz and the +AMF−Rhiz soil treatments, respectively) and that the two proteaceous species would perform better in the −AMF−Rhiz soil treatment. We found that plant root symbionts, specifically treatments with AMF (i.e., +AMF+Rhiz, +AMF−Rhiz), had a significantly positive effect on shoot biomass of the two *Acacia* species, especially *A. acuminata* (effect size = 2.124, *t *=* *0.434, *p *<* *0.01; effect size = 2.619, *t *=* *0.434, *p *<* *0.01, respectively) (Supporting Information Table S2, Figure 1a,b). A similar trend was found for root biomass of both species, especially *A. acuminata* (effect size = 0.869, *t *=* *0.164, *p *<* *0.01; effect size = 1.148, *t *=* *0.164, *p *<* *0.01, respectively) (Supporting Information Table S2, Figure 1e,f) as compared with the treatment with −AMF−Rhiz and no *Bromus* added. This result suggested that AMF has a stronger positive effect on growth of acacias than rhizobia, at least in these microcosms. However, the positive effect of root symbionts on acacia shoot and root biomass became negative if *Bromus* was present, especially for *A. acuminata* (effect size = −1.373, *t *=* *0.614, *p *<* *0.05; effect size = −0.577, *t *=* *0.232, *p *<* *0.05, respectively). This result indicated an overriding negative effect of the invader on acacia biomass (Supporting Information Table S2). For *Eucalyptus astringens* root biomass **+**AMF−Rhiz (effect size = −1.573, *t *=* *0.501, *p *<* *0.05) and −AMF+Rhiz (effect size = −1.148, *t *=* *0.501, *p *<* *0.05) treatments as well as *Bromus* addition (effect size = −1.163, *t *=* *0.501, *p *<* *0.05) to microcosms had a significant negative effect, and this trend, although not significant, was similar for *E. astringens* shoot biomass (Supporting Information Table S2, Figure 2a,b). *Bromus* addition to +AMF+Rhiz treatment also had a negative effect on *C. phoenicus* shoot biomass (effect size = −0.860, *t *=* *0.411, *p *<* *0.05) (Supporting Information Table S2, Figure 3d). Similarly, but without *Bromus* addition, +AMF+Rhiz treatment had a negative effect on both shoot and root biomass of *Eucalyptus loxophleba* (Figure 2d,e), *C. quadrifidus* (Figure 3a,b), *H. lissocarpha* (Supporting Information Table S2, Figure 4d,e). *Hakea prostrata* grew similarly in all soil treatments, irrespective of *Bromus* (Figure 4a,b). Comparably, *Bromus* grew similarly in microcosms irrespective of soil treatment (Supporting Information Table S2, Figure 5a,b).

### 3.2 AMF colonization, root nodules, and root clusters

We hypothesized that AMF and rhizobia will be beneficial to Fabaceae and Myrtaceae in resisting the *Bromus diandrus* invasion, whereas not affect Proteaceae response to invasion in our experimental microcosms. We found that soil treatments with rhizobia (i.e., +AMF+Rhiz and −AMF+Rhiz) had a strong positive effect on the number of root nodules in *A. acuminata*, and the positive effect was stronger in the absence of AMF (effect size = 3.320, *t *=* *1.006, *p *<* *0.05). However, with the addition of *Bromus* to microcosms, the interactive effect of soil treatment and *Bromus* (i.e., −AMF+Rhiz+*Bromus*) had a significantly negative effect on the number of root nodules on *A. acuminata* (effect size = −2.611, *t* = 0.887, *p* < 0.05) (Supporting Information Table S2, Figure 1d). This pattern was reversed for percent AMF colonization of *A. acuminata*, i.e., *Bromus* addition had a significant positive effect on AMF colonization in roots in +AMF+Rhiz treatment (effect size = 2.252, *t* = 0.865, *p* < 0.05) (Supporting Information Table S2, Figure 1c).

Overall, *Acacia microbotrya* had similar number of nodules and AMF colonization in roots across all soil treatments, irrespective of *Bromus* (Supporting Information Table S2, Figure 1g,h). However, the number of root nodules was significantly higher in the +AMF+Rhiz treatment with *Bromus*, suggesting a facilitative effect of the invader on rhizobial nodulation (effect size = 3.509, *t* = 0.720, *p* < 0.01) (Supporting Information Table S2, Figure 1h).

The +AMF+Rhiz treatment had a significantly negative effect on AMF percent colonization in roots of *E. astringens* compared with −AMF−Rhiz (effect size = −15.64, *t* = 6.019, *p* < 0.05) (Supporting Information Table S2, Figure 2c), irrespective of *Bromus* treatment. Similarly, the +AMF+Rhiz treatment had a negative effect on AMF percent colonization of *Bromus* (effect size = −1.175, *t* = 0.187, *p* < 0.01) (Supporting Information Table S2, Figure 5c).

AMF percent colonization in roots of *E. loxophleba* (Figure 2f), *C. quadrifidus* (Figure 3c), and *C. phoenicus* (Figure 3f) was similar irrespective of soil treatment and *Bromus* addition as well as their interactions (Supporting Information Table S2). Similarly, *Hakea prostrata* and *H. lissocarpha* formed a similar number of root clusters irrespective of soil treatment or *Bromus* treatment (Supporting Information Table S2, Figure 4c,f, respectively).

*Acacia acuminata*,* A. microbotrya*,* C. quadrifidus, C. phoenicus*, and *E. loxophleba* had a positive AMF dependency, whereas *E. astringens* and *Bromus* had a negative AMF dependency (Supporting Information Table S2). However, *t*‐tests revealed that these effects were only significant in two cases, i.e., for *A. acuminata* and *A. microbotrya*, suggesting a strong AMF dependency for these two species (Supporting Information Table S3).

Finally, we did not detect AMF in Fabaceae and Myrtaceae roots in the −AMF+Rhiz and −AMF−Rhiz treatments. We did, however, detect one root nodule on each of two *A. acuminata* plants growing in two replicate microcosms of the −AMF−Rhiz treatment; the microcosms were in different blocks, and one nodule was unusually fan‐shaped.

## 4 DISCUSSION

Plant species coexistence is mediated by negative feedbacks that promote cooccurrence of multiple species and ultimately contributes to species richness and ecosystem stability in plant communities (Bever, Platt, & Morton, 2012; Mack & Bever, 2014; Petermann, Fergus, Turnbull, & Schmid, 2008). Soil microorganisms, both beneficial plant root symbionts and plant pathogens, play an important role in mediating plant–soil feedbacks and contribute to ecosystem stability, species diversity as well as ecosystem invasibility (Bever, Mangan, & Alexander, 2015; Callaway et al., 2004; Dawson & Schrama, 2016; Klironomos et al., 2011; Pringle et al., 2009; van der Putten et al., 2013). In this study, our hypothesis was that the presence of arbuscular mycorrhizal fungi and rhizobia would influence the competitive outcomes among woody plant species and mediate the resistance of the native plant communities to weed invasion by *Bromus*.

Our results suggest that AMF and rhizobia provided a competitive advantage (i.e., increased biomass) to the acacias (Fabaceae), had little to no effect on four species of Myrtaceae, and had a negative effect on the growth of *Hakea lissocarpha* (Proteaceae). It is well established that Fabaceae, especially acacias, benefit strongly from AMF and rhizobia (García‐Parisi, Lattanzi, Grimoldi, Druille, & Omacini, 2017; Ossler, Zielinski, & Heath, 2015; Simonsen, Dinnage, Barrett, Prober, & Thrall, 2017). Our results support these studies: the two acacias had significantly larger above‐ and belowground biomass in the presence of both plant root symbionts, but especially in the presence of AMF.

Myrtaceae associate predominantly with ectomycorrhizal fungi (ECM; Lodge, 2000) as well as AMF, and some authors have suggested that eucalypts, in particular, receive more growth and nutritional benefits from ECM than AMF (Kariman, Barker, Finnegan, & Tibbett, 2012; Yuan, Huang, Li, & Christie, 2004). However, other studies have found that for some eucalypts, AMF associations can provide greater benefits during seedling establishment (but see Standish et al., 2007), whereas ECM are more prominent in adult trees (Adams, Reddell, Webb, & Shipton, 2006; Chen, Brundrett, & Dell, 2000). Here, we found, that +AMF+Rhiz treatment had a notable negative effect on AMF percent colonization in *E. astringens* roots. This result suggests that despite the AMF presence in soil inoculum, *E. astringens* had low AMF colonization. ECM tend to be ubiquitous in *Eucalyptus* tree roots (Kariman et al., 2012) and may have been present in our experimental plants; however, we did not quantify them. Taken together, the life‐stage‐dependent shifts in mycorrhizas for Myrtaceae and low interspecific competition for Proteaceae may explain the lack of observed soil treatment effect for these species.

We did not expect the proteaceous species to benefit from access to plant root symbionts because they form cluster roots and thus are not reliant on mycorrhizas or rhizobia for nutrient uptake (Lamont, 2003). Rather, we predicted the proteaceous species would grow bigger in microcosms without plant root symbionts because of a competitive advantage. However, we did not observe this result, perhaps because interspecific competition for resources was weak.

Overall, addition of *Bromus* to microcosms affected the native plant biomass and belowground root symbionts, suggesting a belowground effect of *Bromus*. Notably, for one species, *A. acuminata*, the plant biomass and the number of root nodules were significantly reduced when the microcosms were invaded with *Bromus*, while AMF colonization increased, suggesting an interaction between the invader and both AMF and rhizobia. It is plausible that *A. acuminata* increased its phosphorus acquisition, thus investing more into AMF to compensate for impeded growth in the presence of *Bromus*. In the same treatment, AMF percent colonization in *Bromus* was significantly reduced, suggesting a possible belowground competition between *A. acuminata* and *Bromus* for AMF and access to phosphorus.

*Acacia microbotrya* had larger shoot and root biomass in the soil treatment with AMF but in the absence of rhizobia, irrespective of *Bromus*. Contrary to *A. acuminata*,* Bromus* addition had a strong positive effect on the number of root nodules in *A. microbotrya*. It is plausible that *Bromus* addition to microcosms (at 12 weeks) stimulated a belowground competitive response in *Acacia* that increased their investment in nodules or AMF to facilitate their own growth. It has been proposed that if soil fertility is high, grasses and legumes compete predominantly for light and little for soil nutrients (Eisenhauer & Scheu, 2008). However, if nitrogen (N) is limiting, grasses can benefit from N fixed by legumes, but this interaction may in turn reduce the competitive ability of legumes because grasses sequester a majority of the nitrogen (Munoz & Weaver, 1999; Schwinning & Parsons, 1996; Temperton, Mwangi, Scherer‐Lorenzen, Schmid, & Buchmann, 2007). In our study, *Bromus* did not appear to benefit from N fixed by legumes as its biomass was similar across all soil treatments. It is possible that native *Acacia* species were able to compete with *Bromus* because of extra N_2_ from root nodules.

In conclusion, our study highlights largely functional‐type specific responses of native plants to soil treatments and to *Bromus* addition in the microcosms. AMF and rhizobia influenced the competitive outcomes between Fabaceae, Myrtaceae and Proteacea by facilitating the Fabaceae. Fabaceae rely on these both mutualists for their establishment and growth, whereas Myrtaceae and Proteacea are less or not dependent on AMF and rhizobia for plant growth. Here, we showed that *Bromus* invasion disrupted the mutualisms and altered the belowground dynamics in Fabaceae by affecting nodulation and increasing mycorrhizal colonization (Hale, Lapointe, & Kalisz, 2016).

Our results demonstrate that it is important to study plant competition from belowground as well as aboveground perspectives. In our case, the belowground data highlighted the role of plant root symbionts in mediating interactions among native and invasive plants to influence native plant performance, outcomes that were not apparent in the more easily measured aboveground data. This study provides a rare test of the role of belowground biota in structuring plant communities and supports the idea that soil biota are important in this role. From a restoration perspective, while it is often impractical to track belowground responses, our data suggest that it is important to remain cognisant of the likely interactions occurring belowground even if effects are not apparent aboveground. For example, there could be potentially negative effects of missing soil biota on plant interactions and ultimate restoration outcomes (Lin et al., 2015). A more surprising result was the stimulating effect of *Bromus* on root nodule production in *Acacia microbotrya* and AMF colonization in *A. acuminata*. This result adds yet another possible interaction to the gamut of interactions between native plants, their plant root symbionts and weeds in ecosystems. Our experiment has revealed some interesting and complex belowground dynamics that beg further research.

## CONFLICTS OF INTEREST

None declared.

## AUTHOR'S CONTRIBUTIONS

T.K.M., M.T., R.G.B., and R.J.S. designed the study. T.K.M. and R.J.S. conducted glasshouse work and collected data. C.B. analyzed the data. C.B and R.J.S. wrote the article with contributions from all coauthors.

## DATA ACCESSIBILITY

Data are available from the Dryad Digital Repository: https://doi.org/10.5061/dryad.3m3k724.
