# Supplementary material for: Effect of plant root symbionts on performance of native woody species in competition with an invasive grass in multispecies microcosms
Source: Ecol Evol. 2018 Aug 2;8(17):8652–64. doi: 10.1002/ece3.4397 (PMC6157687; doi:10.1002/ece3.4397)
Supplement: Supplementary file 1 [file ECE3-8-8652-s001.pdf]

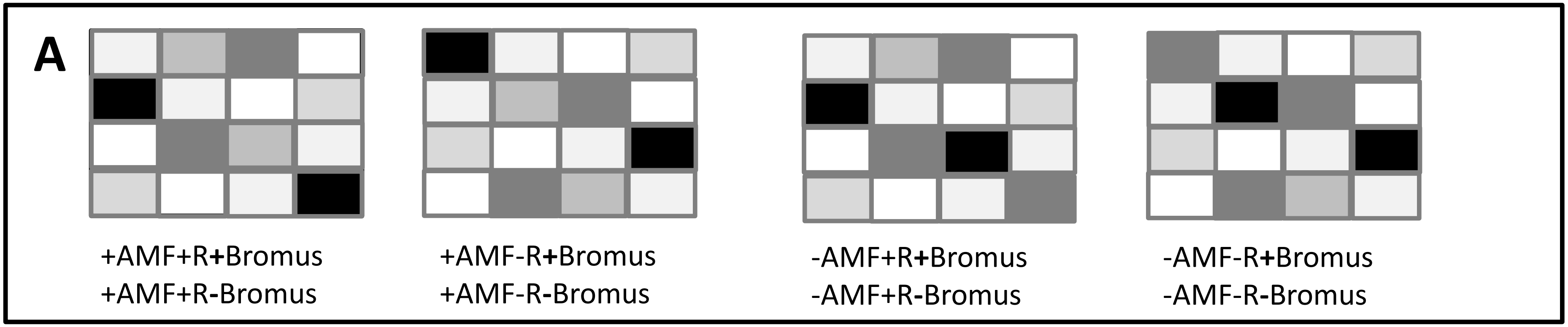
A
+AMF+R+Bromus
+AMF+R-Bromus
+AMF-R+Bromus
+AMF-R-Bromus
-AMF+R+Bromus
-AMF+R-Bromus
-AMF-R+Bromus
-AMF-R-Bromus

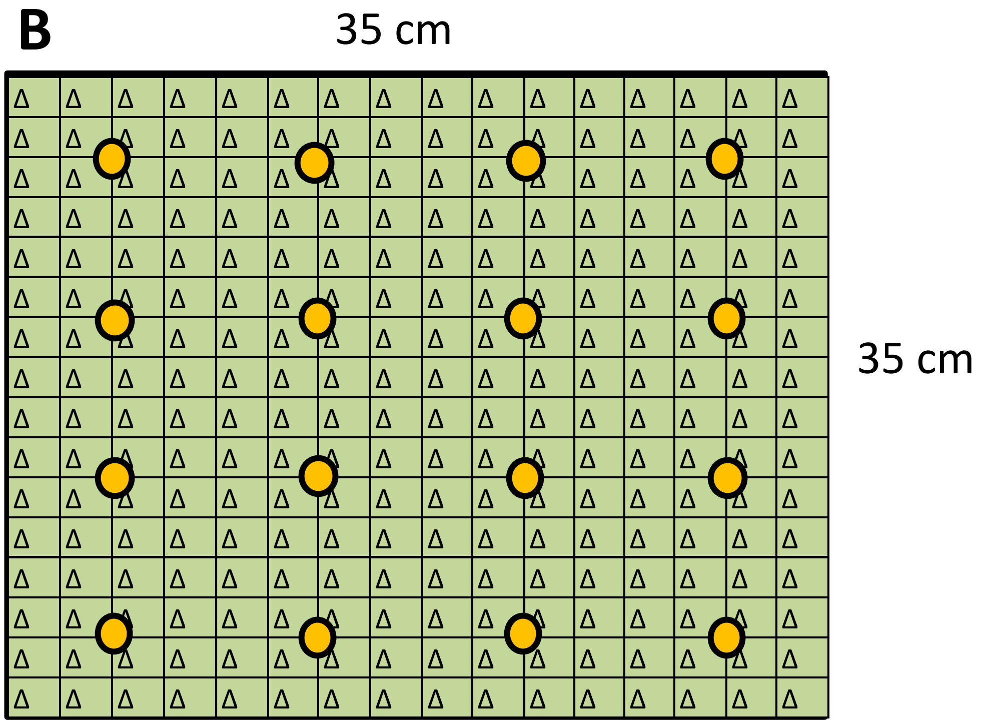
B
35 cm
35 cm
