# Supplementary material for: Effect of plant root symbionts on performance of native woody species in competition with an invasive grass in multispecies microcosms
Source: Ecol Evol. 2018 Aug 2;8(17):8652–64. doi: 10.1002/ece3.4397 (PMC6157687; doi:10.1002/ece3.4397)
Supplement: Supplementary file 2 [file ECE3-8-8652-s002.pdf]

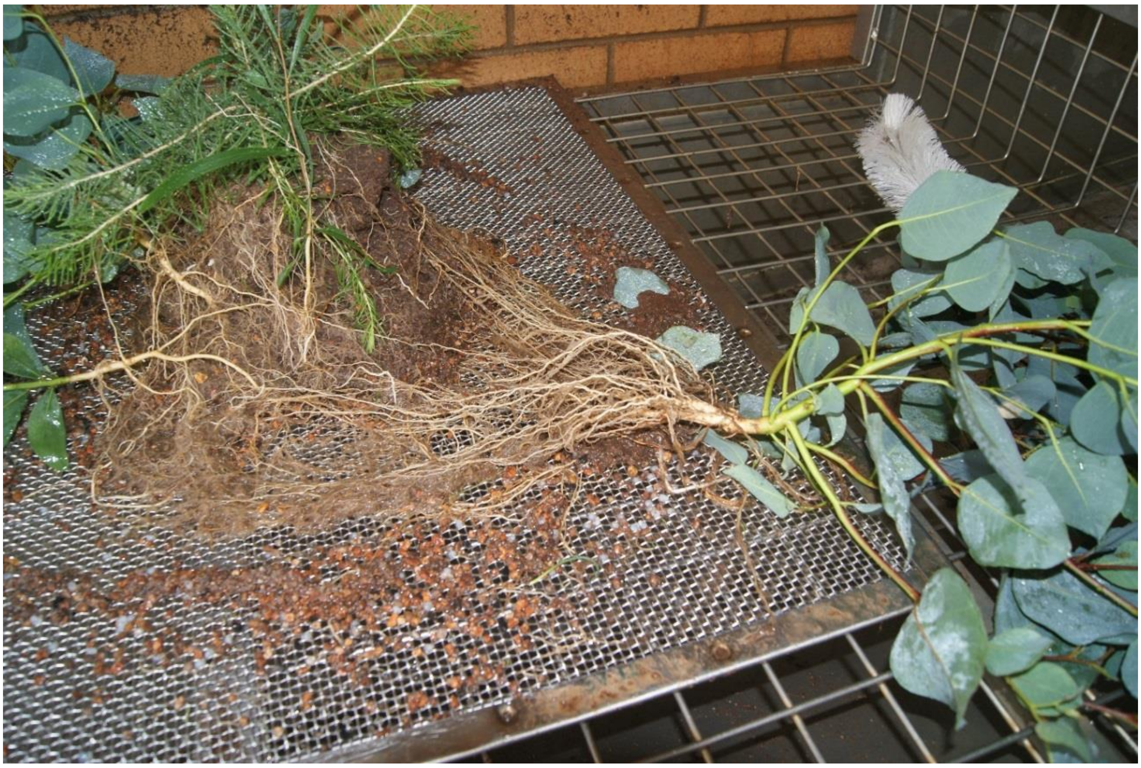

A) Plant roots at harvest. Photo by R. J. Standish.

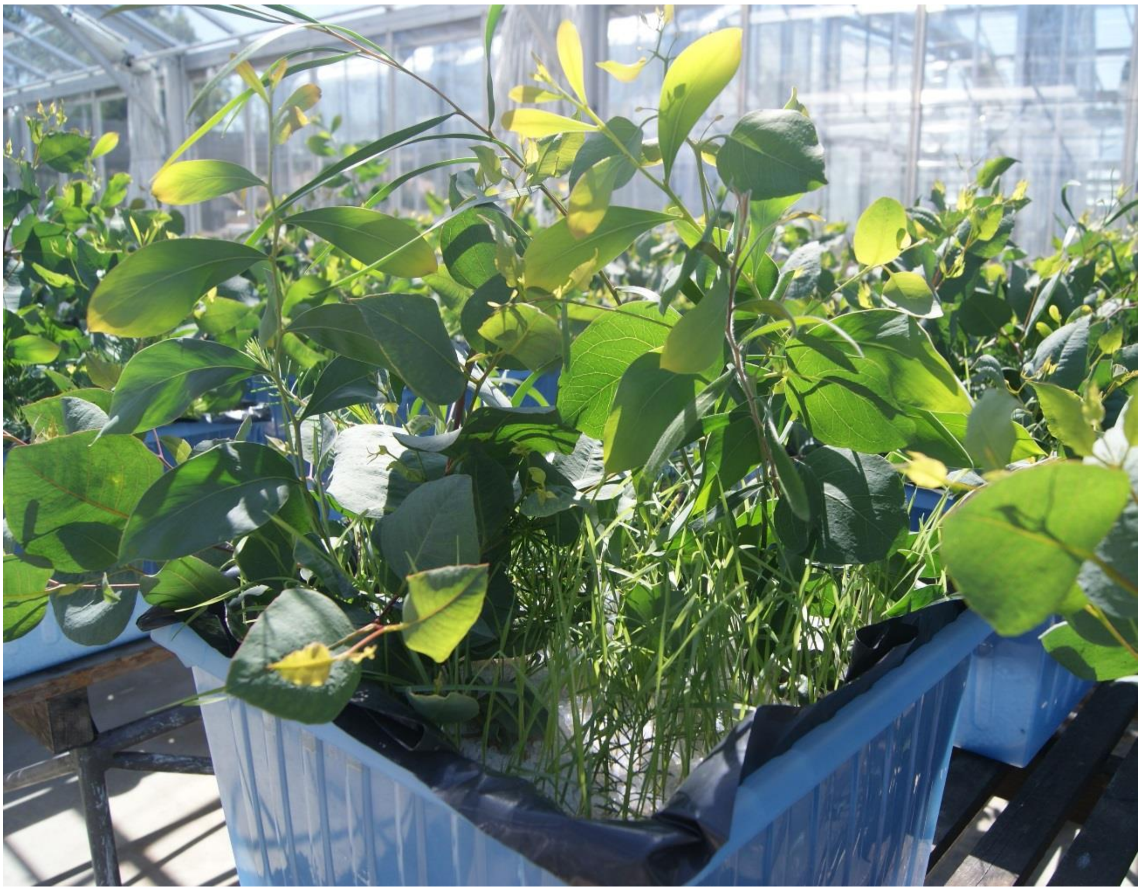

B) Microcosm with *Bromus* ten days prior to harvest. Photo by R. J. Standish.

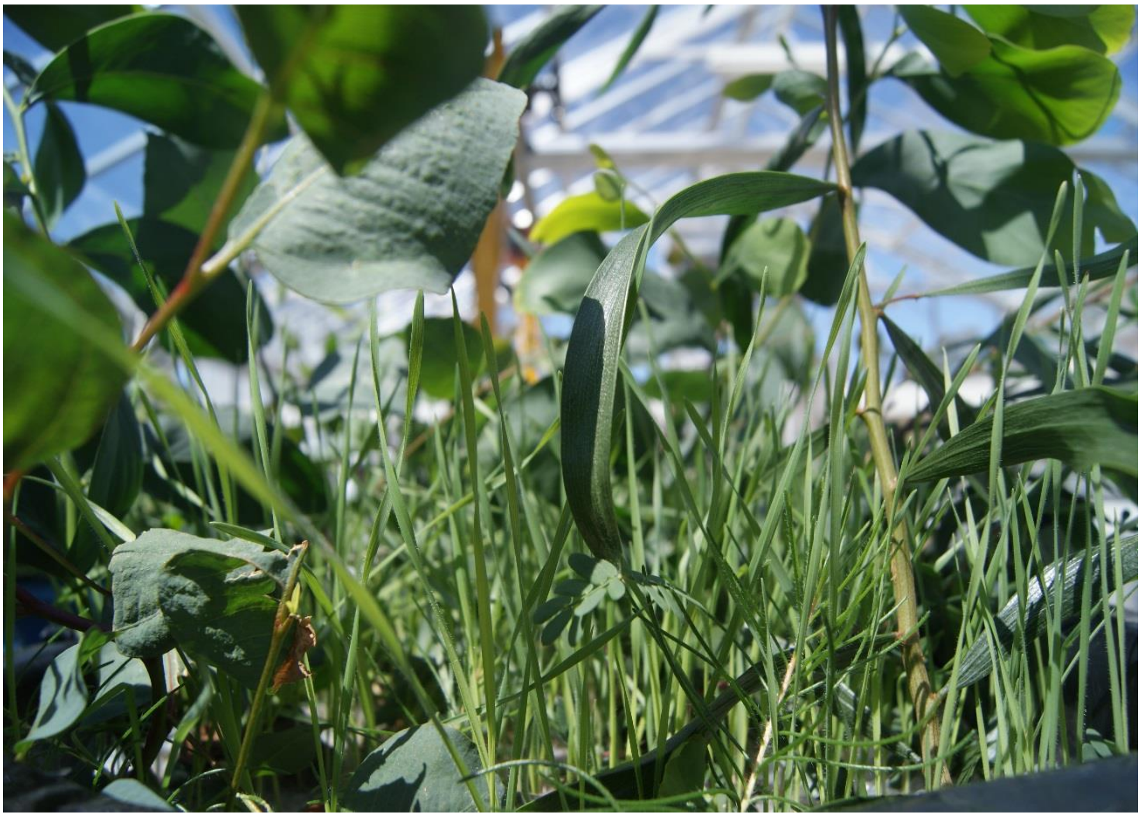

C) Close-up of microcosm with *Bromus* ten days prior to harvest. Photo by R. J. Standish.
