# Supplementary material for: Effect of plant root symbionts on performance of native woody species in competition with an invasive grass in multispecies microcosms
Source: Ecol Evol. 2018 Aug 2;8(17):8652–64. doi: 10.1002/ece3.4397 (PMC6157687; doi:10.1002/ece3.4397)
Supplement: Supplementary file 3 [file ECE3-8-8652-s003.docx]

Supplementary material

**Effect of plant root symbionts on performance of native woody species in competition with an invasive grass in multispecies microcosms**

Running Head: Multispecies interactions in microcosms

Christina Birnbaum^1¶, #a*^, Tim K. Morald^2¶^, Mark Tibbett^3&^, Richard G. Bennett^4&^ and Rachel J. Standish^1,2¶^

^1^Environmental and Conservation Sciences, School of Veterinary and Life Sciences, Murdoch University, 90 South Street, Murdoch, Western Australia 6150, Australia

^2^School of Plant Biology, The University of Western Australia, 35 Stirling Highway, Crawley, Western Australia 6009, Australia

^3^Centre for Agri-Environmental Research, School of Agriculture Policy and Development, University of Reading, Berkshire, RG6 6AR, United Kingdom

^4^Centre for Plant Genetics and Breeding, The University of Western Australia, 35 Stirling Highway, Crawley, Western Australia 6009, Australia

^#a^Current address: Department of Ecology and Evolution, Tulane University, 6823 St. Charles Avenue, New Orleans, Louisiana 70118, United States of America

* Corresponding author

Email: [chbirnbaum@gmail.com](mailto:chbirnbaum@gmail.com), cbirnbaum@tulane.edu (CB)

**Table S1.** **Species traits.** Family, growth form and nutrient acquisition strategies of eight native plant species and *Bromus diandrus* grown in the microcosm experiment. AMF = arbuscular mycorrhizal fungi, ECM = ectomycorrhizal fungi. Inferred for species if reports are available for other species in the genus. AMF associations are visible in stained roots under a microscope, roots colonised by rhizobia develop nodules visible with the naked eye, and cluster roots are bottlebrush-like structures also visible with the naked eye.

| **Species** | **Family** | | **Growth form^a^** | **P acquisition strategy** | **N acquisition strategy** | **References^b^** | |
| --- | --- | --- | --- | --- | --- | --- | --- |
| *A. acuminata* | Fabaceae | Shrub | | AM | Rhizobia | | Standish et al. (2007) |
| *A. microbotrya* | Fabaceae | Shrub | | AM *inferred* | Rhizobia | | NA |
| *C. quadrifidus* | Myrtaceae | Shrub | | AM/ECM *inferred* | Roots | | Warcup 1980 |
| *C. phoeniceus* | Myrtaceae | Shrub | | AM/ECM *inferred* | Roots | | NA |
| *E. loxophleba* | Myrtaceae | Tree | | AM/ECM *inferred* | Roots | | Brundrett et al. (1996) |
| *E. astringens* | Myrtaceae | Tree | | AM/ECM *inferred* | Roots | | NA |
| *H.* *lissocarpha* | Proteaceae | Shrub | | Cluster roots | Roots | | Lamont 1972 |
| *H. prostrata* | Proteaceae | Shrub | | Cluster roots | Roots | | Lamont 1972 |
| *B. diandrus* | Poaceae | Grass | | AM | Roots | | Busby et al. (2013) |

^a^ Growth forms from Flora Base (http://florabase.calm.wa.gov.au/, accessed 24 October 2016).

^b^ References for reported evidence on nutrient acquisition strategies, if known, where AM: arbuscular mycorrhiza, ECM: ectomycorrhizal. *Inferred* denotes no known reported records of species-specific mycorrhizal associations.

**Table S2.** **Results of linear mixed effects models on plant performance in experimental microcosms**. Values in bold indicate significant effect of soil treatment. ******P* < 0.05, ** *P* < 0.01, ↓ indicates results for glmer model (i.e., z-values rather than t-values). Data transformations: § - LN transformed, ¥ - log10, ↑ - square root. Two of the 29 models are likely to be statistically significant due to chance.

| **Species** | **Response** | **Soil treatment** | | | **Intercept** | | |
| --- | --- | --- | --- | --- | --- | --- | --- |
|  |  |  | **Estimate** | **SE (t-value)** | **Estimate** | | **SE (t-value)** |
| ***1) A. acuminata*** | Shoot biomass^§^ | +AMF+Rhiz | **2.124**** | **0.434 (4.891)** | **-1.161*** | | **0.380 (-3.05)** |
|  |  | **+**AMF–Rhiz | **2.619**** | **0.434 (6.032)** |  | |  |
|  |  | –AMF+Rhiz | 0.516 | 0.434 (1.189) |  | |  |
|  |  | *Bromus* Trt | **0.928*** | **0.434 (2.138)** |  | |  |
|  |  | (+AMF+Rhiz)**Bromus* | **-1.373*** | **0.614 (-2.236)** |  | |  |
|  |  | (+AMF–Rhiz)**Bromus* | -0.368 | 0.614 (-0.600) |  | |  |
|  |  | (–AMF+Rhiz)**Bromus* | -0.658 | 0.614 (-1.071) |  | |  |
|  |  |  |  |  |  | |  |
|  | Root biomass^§^ | +AMF+Rhiz | **0.869**** | **0.164 (5.292)** | **-1.018*** | | **0.152 (-6.679)** |
|  |  | **+**AMF–Rhiz | **1.148**** | **0.164 (6.993)** |  | |  |
|  |  | –AMF+Rhiz | 0.116 | 0.164 (0.708) |  | |  |
|  |  | *Bromus* Trt | 0.328 | 0.164 (1.999) |  | |  |
|  |  | (+AMF+Rhiz)**Bromus* | **-0.577*** | **0.232 (-2.487)** |  | |  |
|  |  | (+AMF–Rhiz)**Bromus* | -0.160 | 0.232 (-0.693) |  | |  |
|  |  | (–AMF+Rhiz)**Bromus* | -0.166 | 0.232 (-0.718) |  | |  |
|  |  |  |  |  |  | |  |
|  | Number of nodules^↑^ | +AMF+Rhiz | **2.559*** | **0.786 (3.254)** | -1.001 | | 0.939 (-1.065) |
|  |  | –AMF+Rhiz | **3.320*** | **1.006 (3.300)** |  | |  |
|  |  | *Bromus* Trt | **1.995*** | **0.627 (3.180)** |  | |  |
|  |  | (–AMF+Rhiz)**Bromus* | **-2.611*** | **0.887 (-2.943)** |  | |  |
|  |  |  |  |  |  | |  |
|  | % AMF colonization^↑^ | +AMF+Rhiz | **-1.562*** | **0.612 (-2.552)** | **3.383*** | | **0.448 (7.551)** |
|  |  | *Bromus* Trt | **-1.743** | **0.612 (-2.848)** |  | |  |
|  |  | (+AMF+Rhiz)**Bromus* | **2.252*** | **0.865 (2.601)** |  | |  |
|  |  |  |  |  |  | |  |
| ***2) A. microbotrya*** | Shoot biomass^§^ | +AMF+Rhiz | 0.409 | 0.624 (0.657) | 0.127 | | 0.441 (0.290) |
|  |  | **+**AMF–Rhiz | **1.774*** | **0.624 (2.843)** |  | |  |
|  |  | –AMF+Rhiz | -0.565 | 0.624 (-0.906) |  | |  |
|  |  | *Bromus* Trt | 0.379 | 0.624 (0.607) |  | |  |
| **Species** | **Response** | **Soil treatment** | | | **Intercept** | | |
|  |  |  | **Estimate** | **SE (t-value)** | **Estimate** | | **SE (t-value)** |
| ***2) A. microbotrya*** | Shoot biomass^§^ | (+AMF+Rhiz)**Bromus* | 0.224 | 0.882 (0.255) |  | |  |
|  |  | (+AMF–Rhiz)**Bromus* | -0.230 | 0.882 (-0.261) |  | |  |
|  |  | (–AMF+Rhiz)**Bromus* | -0.315 | 0.882 (-0.357) |  | |  |
|  |  |  |  |  |  | |  |
|  | Root biomass^§^ | +AMF+Rhiz | 0.261 | 0.626 (0.417) | -0.642 | | 0.442 (-1.451) |
|  |  | **+**AMF–Rhiz | **1.624*** | **0.626 (2.595)** |  | |  |
|  |  | –AMF+Rhiz | -0.851 | 0.626 (-1.360) |  | |  |
|  |  | *Bromus* Trt | 0.048 | 0.626 (0.078) |  | |  |
|  |  | (+AMF+Rhiz)**Bromus* | 0.189 | 0.885 (0.214) |  | |  |
|  |  | (+AMF–Rhiz)**Bromus* | 0.113 | 0.885 (0.129) |  | |  |
|  |  | (–AMF+Rhiz)**Bromus* | -0.077 | 0.885 (-0.087) |  | |  |
|  |  |  |  |  |  | |  |
|  | Number of nodules^↓^ | +AMF+Rhiz | 0.476 | 0.424 (1.124) | 0.624 | | 0.602 (1.036) |
|  |  | *Bromus* Trt | -1.098 | 0.666 (-1.648) |  | |  |
|  |  | (+AMF+Rhiz)**Bromus* | **3.509**** | **0.720 (4.868)** |  | |  |
|  |  |  |  |  |  | |  |
|  | % AMF colonization^¥^ | +AMF+Rhiz | -0.120 | 0.182 (-0.661) | **1.148**** | | **0.129 (8.884)** |
|  |  | *Bromus* Trt | 0.274 | 0.182 (1.502) |  | |  |
|  |  | (+AMF+Rhiz)**Bromus* | -0.006 | 0.258 (-0.024) |  | |  |
|  |  |  |  |  |  | |  |
| ***3) E. astringens*** | Shoot biomass | +AMF+Rhiz | -3.021 | 7.067 (-0.487) | **27.672** | | **6.148 (4.501)**** |
|  |  | **+**AMF–Rhiz | -11.970 | 7.067 (-1.694) |  | |  |
|  |  | –AMF+Rhiz | -1.228 | 7.067 (-1.694) |  | |  |
|  |  | *Bromus* Trt | -3.021 | 7.067 (-0.427) |  | |  |
|  |  | (+AMF+Rhiz)**Bromus* | 12.659 | 9.995 (1.267) |  | |  |
|  |  | (+AMF–Rhiz)**Bromus* | 3.849 | 9.995 (0.385) |  | |  |
|  |  | (–AMF+Rhiz)**Bromus* | 5.280 | 9.995 (0.5280 |  | |  |
|  |  |  |  |  |  | |  |
|  | Root biomass | +AMF+Rhiz | -0.600 | 0.501 (-1.199) | **2.571**** | | **0.419 (6.124)** |
| **Species** | **Response** | **Soil treatment** | | | **Intercept** | | |
|  |  |  | **Estimate** | **SE (t-value)** | **Estimate** | | **SE (t-value)** |
| ***3) E. astringens*** | Root biomass | **+**AMF–Rhiz | **-1.573*** | **0.501 (-3.142)** |  | |  |
|  |  | –AMF+Rhiz | **-1.148*** | **0.501 (-2.293)** |  | |  |
|  |  | *Bromus* Trt | **-1.163*** | **0.501 (-2.323)** |  | |  |
|  |  | (+AMF+Rhiz)**Bromus* | 1.360 | 0.708 (1.920) |  | |  |
|  |  | (+AMF–Rhiz)**Bromus* | 1.236 | 0.708 (1.746) |  | |  |
|  |  | (–AMF+Rhiz)**Bromus* | 1.450 | 0.708 (2.048) |  | |  |
|  |  |  |  |  |  | |  |
|  | % AMF colonization | +AMF+Rhiz | **-15.64*** | **6.019 ( -2.598)** | **24.193**** | | **4.256 (5.684)** |
|  |  | *Bromus* Trt | -0.376 | 6.019 (-0.063) |  | |  |
|  |  | (+AMF+Rhiz)**Bromus* | 5.583 | 8.512 (0.656) |  | |  |
|  |  |  |  |  |  | |  |
| ***4) E.loxophleba*** | Shoot biomass^§^ | +AMF+Rhiz | -0.766 | 0.688 (-1.113) | **2.029**** | | **0.508 (3.991)** |
|  |  | **+**AMF–Rhiz | -0.013 | 0.688 (-0.020) |  | |  |
|  |  | –AMF+Rhiz | 0.071 | 0.688 (0.104) |  | |  |
|  |  | *Bromus* Trt | 0.375 | 0.746 (0.503) |  | |  |
|  |  | (+AMF+Rhiz)**Bromus* | -0.477 | 1.015 (-0.470) |  | |  |
|  |  | (+AMF–Rhiz)**Bromus* | -0.541 | 1.015 (-0.5340 |  | |  |
|  |  | (–AMF+Rhiz)**Bromus* | -1.042 | 1.015 (-1.026) |  | |  |
|  |  |  |  |  |  | |  |
|  | Root biomass^§^ | +AMF+Rhiz | -0.937 | 0.851 (-1.101) | 0.266 | | 0.622 (0.428) |
|  |  | **+**AMF–Rhiz | -0.141 | 0.851 (-0.165) |  | |  |
|  |  | –AMF+Rhiz | 0.004 | 0.851 (0.006) |  | |  |
|  |  | *Bromus* Trt | -0.062 | 0.922 (-0.068) |  | |  |
|  |  | (+AMF+Rhiz)**Bromus* | 0.042 | 1.254 (0.034) |  | |  |
|  |  | (+AMF–Rhiz)**Bromus* | -0.211 | 1.254 (-0.168) |  | |  |
|  |  | (–AMF+Rhiz)**Bromus* | -1.064 | 1.254 (-0.849) |  | |  |
|  |  |  |  |  |  | |  |
|  | % AMF colonization | +AMF+Rhiz | 1.398 | 4.029 (0.347) | **11.369**** | | **2.849 (3.991)** |
|  |  | *Bromus* Trt | 2.263 | 4.029 (0.562) |  | |  |
| **Species** | **Response** | **Soil treatment** | | | **Intercept** | | |
|  |  |  | **Estimate** | **SE (t-value)** | **Estimate** | | **SE (t-value)** |
| ***4) E.loxophleba*** | % AMF colonization | (+AMF+Rhiz)**Bromus* | -8.764 | 5.697 (-1.538) |  | |  |
|  |  |  |  |  |  | |  |
| ***5) C. quadrifidus*** | Shoot biomass^¥^ | +AMF+Rhiz | -0.139 | 0.258 (0.420) | **0.463*** | | **0.182 (2.536)** |
|  |  | **+**AMF–Rhiz | 0.108 | 0.258 (0.420) |  | |  |
|  |  | –AMF+Rhiz | 0.257 | 0.258 (0.996) |  | |  |
|  |  | *Bromus* Trt | -0.043 | 0.258 (-0.168) |  | |  |
|  |  | (+AMF+Rhiz)**Bromus* | 0.353 | 0.365 (0.967) |  | |  |
|  |  | (+AMF–Rhiz)**Bromus* | -0.166 | 0.365 (-0.455) |  | |  |
|  |  | (–AMF+Rhiz)**Bromus* | -0.102 | 0.365 (-0.280) |  | |  |
|  |  |  |  |  |  | |  |
|  | Root biomass^§^ | +AMF+Rhiz | 0.458 | 0.556 (0.823) | **-1.909** | | **0.393 (-4.852)** |
|  |  | **+**AMF–Rhiz | 0.675 | 0.556 (1.213) |  | |  |
|  |  | –AMF+Rhiz | 0.931 | 0.556 (1.673) |  | |  |
|  |  | *Bromus* Trt | 0.366 | 0.556 (0.658) |  | |  |
|  |  | (+AMF+Rhiz)**Bromus* | 0.069 | 0.787 (0.088) |  | |  |
|  |  | (+AMF–Rhiz)**Bromus* | -0.837 | 0.787 (-1.064) |  | |  |
|  |  | (–AMF+Rhiz)**Bromus* | -0.816 | 0.787 (-1.037) |  | |  |
|  |  |  |  |  |  | |  |
|  | % AMF colonization^¥^ | +AMF+Rhiz | 0.212 | 0.196 (1.083) | **1.155**** | | **0.138 (8.336)** |
|  |  | *Bromus* Trt | -0.057 | 0.196 (-0.291) |  | |  |
|  |  | (+AMF+Rhiz)**Bromus* | -0.388 | 0.277 (-1.402) |  | |  |
|  |  |  |  |  |  | |  |
| ***6) C. phoenicus*** | Shoot biomass^¥^ | +AMF+Rhiz | 0.561 | 0.291 (1.927) | -0.396 | | 0.205 (-1.927) |
|  |  | **+**AMF–Rhiz | 0.371 | 0.291 (1.275) |  | |  |
|  |  | –AMF+Rhiz | 0.475 | 0.291 (1.634) |  | |  |
|  |  | *Bromus* Trt | **0.723*** | **0.291 (2.487)** |  | |  |
|  |  | (+AMF+Rhiz)**Bromus* | **-0.860*** | **0.411 (-2.090)** |  | |  |
|  |  | (+AMF–Rhiz)**Bromus* | -0.424 | 0.411 (-1.031) |  | |  |
| **Species** | **Response** | **Soil treatment** | | | **Intercept** | | |
|  |  |  | **Estimate** | **SE (t-value)** | **Estimate** | | **SE (t-value)** |
| ***6) C. phoenicus*** | Shoot biomass^¥^ | (–AMF+Rhiz)**Bromus* | -0.593 | 0.411 (-1.441) |  | |  |
|  |  |  |  |  |  | |  |
|  | Root biomass^§^ | +AMF+Rhiz | 1.110 | 0.632 (1.757) | **-2.827** | | **0.447 (-6.323)** |
|  |  | **+**AMF–Rhiz | 0.386 | 0.632 (0.611) |  | |  |
|  |  | –AMF+Rhiz | 1.152 | 0.632 (1.822) |  | | |
|  |  | *Bromus* Trt | 1.062 | 0.632 (1.681) |  | |  |
|  |  | (+AMF+Rhiz)**Bromus* | -1.475 | 0.894 (-1.650) |  | |  |
|  |  | (+AMF–Rhiz)**Bromus* | -0.442 | 0.894 (-0.495) |  | |  |
|  |  | (–AMF+Rhiz)**Bromus* | -1.290 | 0.894 (-1.443) |  | |  |
|  |  |  |  |  |  | |  |
|  | % AMF colonization^§^ | +AMF+Rhiz | -0.398 | 0.463 (-0.858) | **3.368**** | | **0.328 (10.26)** |
|  |  | *Bromus* Trt | -0.392 | 0.463 (-0.847) |  | |  |
|  |  | (+AMF+Rhiz)**Bromus* | 0.244 | 0.656 (0.372) |  | |  |
|  |  |  |  |  |  | |  |
| ***7) H. prostrata*** | Shoot biomass | +AMF+Rhiz | 0.136 | 0.576 (0.236) | **1.867**** | | **0.444 (4.199)** |
|  |  | **+**AMF–Rhiz | -0.042 | 0.576 (-0.074) |  | |  |
|  |  | –AMF+Rhiz | 0.1263 | 0.576 (0.219) |  | |  |
|  |  | *Bromus* Trt | -0.220 | 0.576 (-0.382) |  | |  |
|  |  | (+AMF+Rhiz)**Bromus* | 0.137 | 0.815 (-0.377) |  | |  |
|  |  | (+AMF–Rhiz)**Bromus* | 0.366 | 0.815 (0.449) |  | |  |
|  |  | (–AMF+Rhiz)**Bromus* | -0.307 | 0.815 (-0.377) |  | |  |
|  |  |  |  |  |  | |  |
|  | Root biomass | +AMF+Rhiz | 0.101 | 0.112 (0.908) | **0.364 **** | | **0.081 (4.447)** |
|  |  | **+**AMF–Rhiz | 0.102 | 0.112 (0.914) |  | |  |
|  |  | –AMF+Rhiz | -0.035 | 0.112 (-0.312) |  | |  |
|  |  | *Bromus* Trt | -0.121 | 0.112 (-1.081) |  | |  |
|  |  | (+AMF+Rhiz)**Bromus* | 0.037 | 0.158 (0.236) |  | |  |
|  |  | (+AMF–Rhiz)**Bromus* | 0.056 | 0.158 (0.359) |  | |  |
|  |  | (–AMF+Rhiz)**Bromus* | 0.050 | 0.158 (0.315) |  | | |
| **Species** | **Response** | **Soil treatment** | | | **Intercept** | | |
|  |  |  | **Estimate** | **SE (t-value)** | **Estimate** | | **SE (t-value)** |
| ***7) H. prostrata*** | Number of clusters | +AMF+Rhiz | 1.875 | 4.540 (0.413) | **13.625**** | | **3.237 (4.209)** |
|  |  | **+**AMF–Rhiz | -1.250 | 4.540 (-0.275) |  | |  |
|  |  | –AMF+Rhiz | -4.750 | 4.540 (-0.740) |  | |  |
|  |  | *Bromus* Trt | -3.125 | 4.540 (-0.688) |  | |  |
|  |  | (+AMF+Rhiz)**Bromus* | 3.125 | 6.420 (0.487) |  | |  |
|  |  | (+AMF–Rhiz)*Bromus | 1.125 | 6.420 (0.175) |  | |  |
|  |  | (+AMF–Rhiz)**Bromus* | 1.125 | 6.420 (0.175) |  | |  |
|  |  | (–AMF+Rhiz)**Bromus* | -4.750 | 6.420 (-0.740) |  |  | |
|  |  |  |  |  |  | |  |
| ***8) H. lissocarpha*** | Shoot biomass | +AMF+Rhiz | **-2.088*** | **0.714 (-2.924)** | **3.182**** | | **0.612 (5.196)** |
|  |  | **+**AMF–Rhiz | -1.072 | 0.714 (-1.501) |  | |  |
|  |  | –AMF+Rhiz | -1.215 | 0.714 (-1.701) |  | |  |
|  |  | *Bromus* Trt | -0.541 | 0.714 (-0.758) |  | |  |
|  |  | (+AMF+Rhiz)**Bromus* | 2.070 | 1.010 (2.049) |  | |  |
|  |  | (+AMF–Rhiz)**Bromus* | 0.756 | 1.010 (0.749) |  | |  |
|  |  | (–AMF+Rhiz0**Bromus* | 1.592 | 1.010 (1.576) |  | |  |
|  |  |  |  |  |  | |  |
|  | Root biomass | +AMF+Rhiz | **-0.209*** | **0.100 (-2.090)** | **0.344**** | | **0.076 (4.519)** |
|  |  | **+**AMF–Rhiz | -0.008 | 0.100 (-0.081) |  | |  |
|  |  | –AMF+Rhiz | -0.124 | 0.100 (-1.241) |  | |  |
|  |  | *Bromus* Trt | -0.075 | 0.100 (-0.755) |  | |  |
|  |  | (+AMF+Rhiz)**Bromus* | 0.239 | 0.141 (1.689) |  | |  |
|  |  | (+AMF–Rhiz)**Bromus* | 0.047 | 0.141 (0.335) |  | |  |
|  |  | (–AMF+Rhiz)**Bromus* | 0.099 | 0.141 (0.701) |  | |  |
|  |  |  |  |  |  | |  |
|  | Number of clusters | +AMF+Rhiz | -12.500 | 7.585 (-1.648) | **26.125 **** | | **6.442 (4.056)** |
|  |  | **+**AMF–Rhiz | -3.375 | 7.585 (-0.445) |  | |  |
|  |  |  | | |  | | |
|  |  |  | | |  | | |
|  |  |  | | |  | | |
| **Species** | **Response** | **Soil treatment** | | | **Intercept** | | |
|  |  |  | **Estimate** | **SE (t-value)** | **Estimate** | | **SE (t-value)** |
| ***8) H. lissocarpha*** | Number of clusters | –AMF+Rhiz | -7.500 | 7.585 (-0.989) |  | |  |
|  |  | *Bromus* Trt | 2.000 | 7.585 (0.264) |  | |  |
|  |  | (+AMF+Rhiz)**Bromus* | 1.250 | 10.727 (0.117) |  | |  |
|  |  | (+AMF–Rhiz)**Bromus* | -5.875 | 10.727 (-0.548) |  | |  |
|  |  | (–AMF+Rhiz)**Bromus* | 3.250 | 10.727 (0.303) |  | |  |
|  |  |  |  |  |  | |  |
| ***9) B.diandrus*** | Shoot biomass | +AMF+Rhiz | -4.665 | 3.696 (-1.262) | **10.512**** | | **2.614 (4.022)** |
|  |  | **+**AMF–Rhiz | -2.830 | 3.696 (-0.766) |  | |  |
|  |  | –AMF+Rhiz | -1.402 | 3.696 (-0.379) |  | |  |
|  |  |  |  |  |  | |  |
|  | Root biomass | +AMF+Rhiz | -1.313 | 1.021(-1.286) | **3.514**** | | **0.722 (4.866)** |
|  |  | **+**AMF–Rhiz | 0.195 | 1.021 (0.191) |  | |  |
|  |  | –AMF+Rhiz | 0.435 | 1.021 (0.426) |  | |  |
|  |  |  |  |  |  | |  |
|  | % AMF colonization^↓^ | +AMF+Rhiz | **-1.175**** | **0.187 (-6.280)** | **3.309**** | | **0.509 (6.493)** |
|  |  |  |  |  |  | |  |

**Table S3. Shoot biomass (SB) and mycorrhizal dependency (MD).** SB data are means ± SE. Values in bold indicate statistically significant differences between groups. AMF – arbuscular mycorrhizal fungi, SDW – shoot dry weight, MD – mycorrhizal dependency. Values in bold indicate significant differences between groups (P < 0.05).

| Species | SDW (g plant^-1^) | | *t-value* | *P- value* | MD (%) |
| --- | --- | --- | --- | --- | --- |
|  | With AMF | Without AMF |  |  |  |
| *A. acuminata* | 4.35 ± 0.48 | 0.42 ± 0.14 | **-7.78** | **< 0.001** | 90.30 |
| *A. microbotrya* | 6.83 ± 1.08 | 1.43 ± 0.42 | **-4.66** | **0.001** | 78.96 |
| *C. quadrifidus* | 4.52 ± 1.27 | 3.37 ± 1.15 | -0.66 | 0.51 | 25.38 |
| *C. phoeniceus* | 1.12 ± 0.24 | 0.62 ± 0.64 | -1.48 | 0.16 | 44.39 |
| *E. loxophleba* | 9.67 ± 3.04 | 8.21 ± 1.89 | -0.40 | 0.68 | 15.15 |
| *E. astringens* | 15.70 ± 6.9 | 27.67 ± 2.67 | 1.61 | 0.14 | -76.23 |
| *B. diandrus* | 7.68 ± 1.26 | 10.51 ± 3.92 | 0.68 | 0.53 | -36.84 |

**Fig S1.** **Microcosms.** A) Four of 32 random planting designs with one of four soil treatments and two *Bromus* treatments (+Bromus, –Bromus) indicated below each design. AMF = arbuscular mycorhizzal fungi; R = rhizobia. Each microcosm contained 16 seedlings (two of each species). B) Close-up of a microcosm showing 256 *Bromus diandrus* (triangles) and 16 native seedlings (yellow-filled circles) (PDF).

**Fig S2. Harvest.** A) Plant roots at harvest. B) Microcosm with *Bromus* ten days prior to harvest. C) Close-up of microcosm with *Bromus* ten days prior to harvest. (PDF)
